# The Future of Midwifery Practice

**Published:** 1898-11-19

**Authors:** 


### The Future of Midwifery Practice.

We pointed out last week that, in consequence of the action of the General Medical Council in regard to the employment of unqualified assistants, a good deal of the midwifery which has hitherto been attended, not perhaps personally by medical men, but at any rate by assistants directly responsible to them, would in future fall into the hands of midwives acting entirely independently, unless the Medical Council should think fit to sanction the employment of midwives by medical practitioners to attend ordinary confinements. We do not say that this would be an ideal plan. The ideal system would be, no doubt, that every woman should be able to obtain the assistance of a fully qualified medical practitioner at the time of her confinement, and that the practice of midwifery by any except such fully qualified practitioners should be absolutely suppressed by law. But we do not think that any such suggestion comes within the scope of practical politics. What we have to deal with is the actual, and what is actually happening is that a very large number of women, more or less trained, are being turned out every year to earn their living by the practice of midwifery, that even now they are within their rights in so practising, and that there is every probability that within a few years they will, by licence or otherwise, obtain some form of legal recognition. The question then that we have to consider is which is best for them, for the patients, and for ourselves; that these midwives should "practice" independently, sending for such help as they may happen to be able to procure when they get into difficulties, or that they should be employed as midwifery assistants by general medical practitioners, under whose orders they should act in all cases. We can have no hesitation in saying that the latter plan would be infinitely the best. The only thing that stands in the way is that those who have the fear of the General Medical Council before their eyes, *i.e.*, the best of the practitioners, hesitate to take a step which, for aught they can tell, might end in their being turned off the Register. We think that this state of uncertainty ought to be put an end to, and that the General Medical Council ought to state what it is prepared to recognise as the "legitimate" employment of midwives. The importance of the question is very strongly brought home to us by what we see going on around us on every side. Among the women who are now taking up midwifery are many who are quick to perceive how readily the "training," which can be picked up in a few months, may be turned to commercial use; and what is happening is, that as, pending some pronouncement by the Medical Council, the doctors dare not employ the midwives, the midwives are employing the doctors. In the recently issued Transactions of the Midwives Society, one of these ladies, in relating how she "proposed to have the patient anæsthetised," goes on to say that "a medical man in whom I felt reliance was sent for," and "my medical friend administering the chloroform," &c.

We need not multiply instances, but to show more precisely the drift of affairs we would refer to a short article, which appeared in the nursing section of The Hospital a few weeks ago, giving a description of a maternity which had been established in one of the London suburbs by a midwife, who not only gets fees from her "patients," but also from the pupils whom she trains in midwifery work. All these pupils are said to see plenty of cases. The "organiser and owner" gives "a weekly lecture on the theoretical part of the work, whilst a doctor resident in the vicinity gives another." The proprietor "charges her patients according to their means; when anyone can afford to pay £1 1s. she will not nurse them for less. In the event of medical aid being required she shares this fee with the doctor called in, and is responsible that he is paid." Clearly, the midwife is the employer. And this is what the practice of midwifery tends to if it is allowed to drift out of the hands of the medical profession. Needless to add that, although the maternity to which we have alluded is only a small affair at present, "the work has grown so enormously that in the spring the owner will remove to a large and convenient house which she is having built for the purpose!" No private practice conducted by medical men or medical women who have had to undergo a long and expensive education can compete with such a system. Medical practitioners, however, could easily hold their own if they could employ midwives, while the public would gain the inestimable advantage of being able to look to some responsible head when midwives are employed.

